# Telemedicine in Malignant and Nonmalignant Hematology: Systematic Review of Pediatric and Adult Studies

**DOI:** 10.2196/29619

**Published:** 2021-07-08

**Authors:** Aashaka C Shah, Linda C O’Dwyer, Sherif M Badawy

**Affiliations:** 1 Department of Medical Education University of Illinois College of Medicine Chicago, IL United States; 2 Galter Health Sciences Library and Learning Center Northwestern University Feinberg School of Medicine Chicago, IL United States; 3 Division of Pediatric Hematology, Oncology, Neuro-Oncology & Stem Cell Transplantation Ann & Robert H Lurie Children's Hospital of Chicago Chicago, IL United States; 4 Department of Pediatrics Northwestern University Feinberg School of Medicine Chicago, IL United States

**Keywords:** telemedicine, telehealth, eHealth, Digital Health, Digital Medicine, mHealth, hematology, malignant, nonmalignant, intervention

## Abstract

**Background:**

Telemedicine, including video-, web-, and telephone-based interventions, is used in adult and pediatric populations to deliver health care and communicate with patients. In the realm of hematology, telemedicine has recently been used to safely and efficiently monitor treatment side-effects, perform consultations, and broaden the reach of subspecialty care.

**Objective:**

We aimed to synthesize and analyze information regarding the feasibility, acceptability, and potential benefits of telemedicine interventions in malignant and nonmalignant hematology, as well as assess the recognized limitations of these interventions.

**Methods:**

Studies were identified through a comprehensive Medical Subject Headings (MeSH) search on the PubMed MEDLINE, Controlled Register of Clinical Trials (Cochrane CENTRAL from Wiley), Embase, and CINAHL (EBSCO) databases on February 7, 2018. A second search, utilizing the same search strategy, was performed on October 1, 2020. We followed the Preferred Reporting Items for Systematic Reviews and Meta-Analyses (PRISMA) guidelines in the reporting of included evidence. Included studies were original articles researching the feasibility, acceptability, and clinical outcomes of telemedicine or telehealth interventions in pediatric or adult populations with malignant or nonmalignant hematological conditions. Data items in the extraction form included first author name, publication year, country, malignant or nonmalignant hematological condition or disease focus of the study, participant age, participant age subgroup (pediatric or adult), study design and setting, telemedicine intervention type and description, study purpose, and main study outcomes.

**Results:**

A total of 32 articles met the preset criteria and were included in this study. Most (25/32) studies were conducted in adults, and the remaining (7/32) were conducted in the pediatric population. Of the 32 studies, 12 studied malignant hematological conditions, 18 studied nonmalignant conditions, and two studied both malignant and nonmalignant conditions. Study types included pilot study (11/32), retrospective study (9/32), randomized controlled trial (6/32), cross-sectional study (2/32), case study (1/32), pre-post study (1/32), noncomparative prospective study (1/32), and prospective cohort study (1/32). The three main types of telemedicine interventions utilized across all studies were video-based (9/32), telephone-based (9/32), and web-based interventions (14/32). Study results showed comparable outcomes between telemedicine and traditional patient encounter groups across both pediatric and adult populations for malignant and nonmalignant hematological conditions.

**Conclusions:**

Evidence from this review suggests that telemedicine use in nonmalignant and malignant hematology provides similar or improved health care compared to face-to-face encounters in both pediatric and adult populations. Telemedicine interventions utilized in the included studies were well received in both pediatric and adult settings. However, more research is needed to determine the efficacy of implementing more widespread use of telemedicine for hematological conditions.

## Introduction

Globally, we are facing a growing shortage of specialist physicians, coupled with inequity in patient access to quality care [1-3]. In America alone, only 30 specialists practice for every 100,000 people living in rural communities compared to 263 specialists in urban environments [4]. This shortage is further exacerbated when examined by subspecialty, making it progressively more difficult for patients to reach their physicians and obtain necessary treatments [4]. Thus, health care officials have turned to the field of telemedicine to leverage technological tools with the goal of expanding and optimizing the delivery of medical care [3].

Telemedicine is defined as the use of teleconferencing interventions to provide and deliver health care to patients [5]. Although telemedicine is classified under the broader term, “telehealth,” ambiguity surrounding which forms of technology each category encompasses still exists, and both terms are often used interchangeably [6]. Telemedicine is a subset of telehealth, which is defined as the use of technology in any aspect of health care. Telemedicine is specifically utilized for clinical patient care, not exclusively for research purposes.

Telemedicine has grown in parallel with society’s growing desire for convenience, efficiency, and productivity, and today, frequently used modes of telemedicine include videoconferencing, email, wearable devices, cellular phones, and various mobile apps [3,6]. These new systems of telemedicine are promising for mitigating the current challenges in health care reform, since they offer medical professionals the novel opportunity to extend their presence to settings outside of their immediate reach [3]. In doing so, telemedicine presents health care industries with the potential to provide more cost-effective treatments, support patient self-management, respond to the growing demand for specialists, and uncover avenues for advancing the practice of medicine in underserved areas worldwide [3,6-9]. Furthermore, access to personal and mobile technologies is ubiquitous [10-14], which has provided an opportunity to optimize digital health care delivery approaches, including telemedicine. However, despite these benefits, there remain obstacles to implementing telemedicine in daily practice [15,16]. Barriers, including stable internet access, cost, and patient desire for in-person appointments, must be addressed for telemedicine to reach its full potential [15,16]. Nevertheless, there has been growing evidence to support the utility and clinical applications of various digital approaches for health care delivery, including telemedicine, across pediatric and adult populations with or without chronic medical conditions [17-39], although the cost-effectiveness remains unclear [40,41].

In the field of hematology, recent advances in telemedicine have been used to conduct patient visits, monitor treatment side-effects, and perform consultations [42-45]. During the current COVID-19 pandemic, telemedicine use in outpatient settings, such as hematology clinics, has increased significantly owing to stay-at-home orders and efforts to lessen exposure to ill patients [46,47]. Videoconferencing interventions have proved to be a safe and efficient way for health care providers and patients to continue managing and monitoring chronic health conditions, especially for sickle cell disease and other hematological conditions [48]. Additionally, both adult and pediatric populations in underserved areas have benefitted from telemedicine’s ability to connect them to specialists in different cities [48].

In this systematic review, we evaluated the evidence available in the literature to analyze the feasibility, acceptability, and potential benefits of telemedicine interventions in malignant and nonmalignant hematology and assess the current limitations of utilizing these interventions. Owing to the heterogeneity of the included studies, the methods by which feasibility, accessibility, and clinical outcomes were assessed vary among the studies. In this review, we use these terms broadly to encompass the different interpretations of these objectives. We more specifically define each study’s main objectives in the Results section.

## Methods

### Guideline

We followed the Preferred Reporting Items for Systematic Reviews and Meta-Analyses (PRISMA) guidelines for reporting of evidence across the studies we reviewed [49].

### Article Retrieval

A librarian, in collaboration with other review authors, collaboratively developed the highly sensitive Medical Subject Headings (MeSH) term–based search strategies and ran searches in the following databases: PubMed MEDLINE, Controlled Register of Clinical Trials (Cochrane CENTRAL from Wiley), Embase, and CINAHL (EBSCO), on February 7, 2018 (Multimedia Appendix 1). The bibliographies of hand-searched articles that had been previously identified were also included. The search strategy focused on articles that studied telemedicine and telehealth interventions for malignant and nonmalignant hematological conditions in both pediatric and adult populations. No date limits were applied to the search. On October 1, 2020, another literature search was conducted on PubMed to identify articles that were published since the last literature search and that met the inclusion criteria. The second literature search utilized the same search strategy as the first search.

### Study Selection

The inclusion criteria were as follows: (1) studies involving pediatric or adult populations with malignant or nonmalignant hematological conditions, (2) studies involving telemedicine or telehealth interventions, (3) studies that included feasibility, acceptability, and clinical outcomes of the interventions as the primary or secondary outcomes, (4) original research articles, and (5) studies designed as a randomized controlled trial (RCT), cohort study, pilot study, retrospective study, or cross-sectional study. “Telemedicine” and “telehealth” are often used interchangeably, so both terms were relevant to the search despite having different semantic meanings. The exclusion criteria were as follows: (1) studies not related to hematology, (2) studies without a telemedicine intervention, (3) nonclinical research studies, (4) abstracts only or nonoriginal research papers, and (5) studies not in English.

### Data Extraction

A standardized format was used for data extraction. Data items in the extraction form included first author name, publication year, country, malignant or nonmalignant hematological condition or disease focus of the study, participant age, participant age subgroup (adult or pediatric), study design and setting, telemedicine intervention type and description (telephone, remote management, videoconferencing, etc), study purpose, and main outcomes. Two authors coded all included articles individually. Disagreements were resolved by discussion.

### Quality Assessment and Evidence Strength

Studies described in each article were evaluated for the quality of evidence using the GRADE (Grades of Recommendation, Assessment, Development, and Evaluation) approach [50]. This method evaluates four key domains, including consistency, directness, risk for bias, and precision of the evidence. Two authors graded all included articles individually. Disagreements were similarly resolved by discussion, if needed.

## Results

### Literature Search

**Figure 1:**
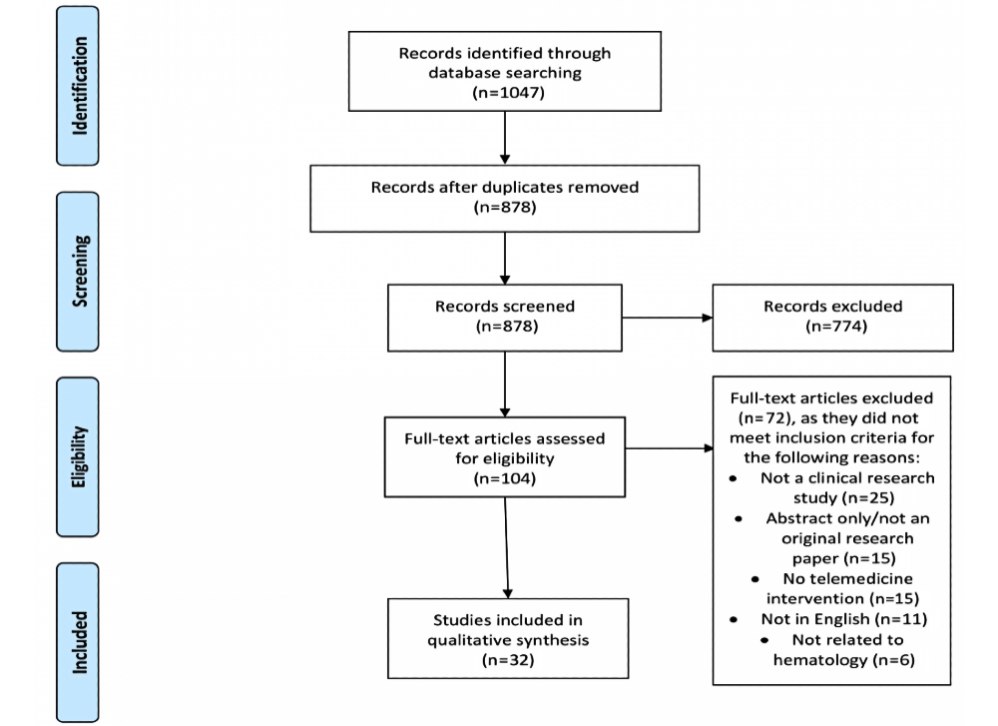
PRISMA (Preferred Reporting Items for Systematic Reviews and Meta-Analyses) flow diagram for the included studies.

Our literature search identified 1047 records. After removing duplicates, 878 articles remained. Two authors independently screened the titles and abstracts of 878 records, and 774 were excluded. Two authors independently screened the remaining 104 full-text articles, and 32 met our inclusion criteria for eligibility. The study flowchart and reasons for exclusion of full-text papers were documented in an adapted PRISMA study flowchart (Figure 1). We did not identify any non-English articles that met our inclusion criteria.

A total of 32 articles were included in this systematic review. Most (25/32, 78%) studies were conducted with adults [42,51-74], while the remaining (7/32, 22%) were conducted in the pediatric population [75-81]. The studies addressed whether a telemedical intervention for patients with malignant and/or nonmalignant hematological conditions is a safe, feasible, and efficacious method of health care provision.

### Description of the Included Studies

Table 1 and Table 2 summarize study characteristics for the pediatric and adult populations, respectively. Out of the 32 studies, 16 (50%) were conducted in the United States [52,53,55-57,60,62,63,65,69,71-73,77,78,81], 6 (19%) in Canada [58,64,68,74,75,79], 2 (6%) each in Australia [42,61], India [51,76], and Italy [54,70], and 1 (3%) each in Brazil [80], France [59], Germany [66], Ireland [67], and Rwanda [81]. Although the search retrieval included results published since 1980, the earliest eligible study was from 1998, which corresponds to our knowledge of when telemedicine was first introduced [71]. The most recent study was published in 2018 [56].

The sample size ranged from 1 [74] to 217,014 [62], with a median of 157 and a mean of 7977 participants per study. Overall, 21 studies enrolled ≥100 participants [51,53-60,62,63,65-70,72,73,76,80] and 11 had <100 participants [42,52,61,64,71,74,75,77-79,81].

Of the 32 included studies, 12 (34%) studied malignant hematological conditions [42,53,58-61,64,67,69,77,80,81], 18 (59%) studied nonmalignant conditions with percentages [51,52,54-57,62,63,65,66,68,70-74,76,78], and 2 (6%) studied both malignant and nonmalignant conditions [75,79].

**Table 1:** Characteristics of pediatric malignant and nonmalignant hematology studies.

Source (country)	Condition	Sample size and age	Study design	Study setting	Grade
Adler 2015 [75] (Canada)	Leukemia, lymphoma, solid tumor, neuro-oncology, and nonmalignant hematology	N=54; age: 0-18 years	Pilot study	The Hospital for Sick Kids (Toronto, Canada) and six telemedicine facilities in Caribbean countries	Very low
Agarwal 2014 [76] (India)	Thalassemia	N=112	Pilot study	Participating centers in Italy, Pakistan, and India	Very low
Cox 2015 [77] (United States)	Survivors of childhood brain tumors or acute lymphoblastic leukemia	N=68; age: 8-16 years	Randomized controlled trial	St. Jude Children’s Research Hospital	Moderate
Jacobson 2016 [78] (United States)	Severe hemophilia	N=12; age: 10-18 years, mean age: 10.25 years	Pilot study	Hemophilia Treatment Center	Very low
Johnston 2017 [79] (Canada)	Any hematology and oncology condition (majority anemia, hemoglobinopathy, and bleeding disorder)	N=85	Cross-sectional study	Champlain BASE e-Consult service (Ottawa, Canada) through a web portal	Low
Pedrosa 2017 [80] (Brazil)	Acute lymphoblastic leukemia	N=163; age: 1-15 years	Pilot study	Instituto Materno Infantil de Pernambuco (Recife, Brazil) and St. Jude Children’s Research Hospital	Very low
Stulac 2016 [81] (United States, Rwanda)	Lymphoma, sarcoma, leukemia, and other malignancies	N=24	Retrospective study	Rural district hospitals in Rwanda (Rwinkwavu, Kirehe, and Butaro)	Very low

**Table 2:** Characteristics of adult malignant and nonmalignant hematology studies.

Source (country)	Condition	Sample size and age	Study design	Study setting	Grade
Agrawal 2014 [51] (India)	Blood transfusion donors	N=16,438 donations	Pilot study	Regional Blood Transfusion Centre (Dehradun, Uttarakhand)	Very low
Applebaum 2012 [52] (United States)	Posttraumatic stress disorder related to hematopoietic stem cell transplantation	N=46	Randomized controlled trial	Memorial Sloan-Kettering Cancer Center, Mount Sinai Medical Center, and Hackensack University Medical Center	Moderate
Bakitas 2015 [53] (United States)	Advanced-stage solid tumor or hematological malignancy with oncologist-determined prognosis of 6-24 months	N=207; early group mean age: 64.03 years, delayed group mean age: 64.6 years	Randomized controlled trial	National Cancer Institute Center and Veterans Affairs Medical Center	Moderate
Barcellona 2013 [54] (Italy)	Thromboembolic conditions	N=114; mean age: 61 years	Pre-post study	Thrombosis centers	Low
Blissit 2015 [55] (United States)	Thromboembolic conditions	N=200; face-to-face group (n=90) and telephone group (n=110)	Retrospective study	Veterans Affairs Medical Center and affiliated rural clinics in South Carolina	Very low
Breen 2015 [42] (Australia)	Hodgkin/non-Hodgkin lymphoma and chronic lymphocytic leukemia	N=18; mean age: 48.4 years	Pilot study	Chemotherapy day unit and hematology inpatient ward	Very low
Burwick 2018 [56] (United States)	Monoclonal gammopathy of undetermined significance	N=152; median age: 69 years	Retrospective study	Veterans Health Administration facilities	Very low
Cecchini 2016 [57] (United States)	Varying hematological conditions (majority anemia, thrombocytopenia, and venous thromboembolism)	N=909; e-consult (n=302), median age: 64 years; face-to-face consult before e-consult (n=305), median age: 69.3 years; face-to-face consult after e-consult (n=302), median age: 65.9 years	Retrospective study	Veterans Affairs Connecticut Healthcare System	Very low
Clarke 2011 [58] (Canada)	Cancer (majority gastrointestinal and lymphoma)	N=712	Retrospective study	48 Local Health Areas (British Columbia, Canada)	Very low
Compaci 2011 [59] (France)	Diffuse large B-cell lymphoma	N=100; median age: 57 years	Noncomparative prospective study	Toulouse University Hospital	Very low
Flannery 2009 [60] (United States)	Medical oncology and hematologic malignant diagnoses	N=5283; mean age 61.1 years	Descriptive retrospective study	University of Rochester Medical Center and James P. Wilmot Cancer Center	Very low
Hung 2014 [61] (Australia)	Hematological malignancies in patients who received peripheral blood stem cell transplants	N=37; usual care (n=19), age: 59.9 years; extended care (n=18), age: 57.5 years	Randomized controlled trial	The Hematology and Oncology Clinics of Australia, The Wesley Hospital (Brisbane, Australia)	Low
Kirsh 2015 [62] (United States)	Hematology and other specialties	N=217,014	Pilot study	Veterans Health Administration and Veterans Integrated Service Networks	Very low
Najafi 2017 [63] (United States)	Hematology and other specialties	N=313 (63 hematology)	Pilot study	University of California San Francisco Hospital in Mission Bay	Very low
Overend 2008 [64] (Canada)	Indolent and chronic hematological malignancies	N=53; median age: 66.5 years	Pilot study	British Columbia Cancer Agency’s Centre for the Southern Interior in Kelowna	Very low
Philip 2015 [65] (United States)	Thromboembolic conditions	N=502	Pilot study	Harris Health System (Houston, Texas)	Very low
Prochaska 2017 [66] (Germany)	Thromboembolic conditions in patients receiving vitamin K antagonists	N=2221; median age: 73 years	Prospective cohort study	Center of Thrombosis and Hemostasis, University Medical Center Mainz	Very low
Reid 2011 [67] (Ireland)	Cancer	N=7498	Retrospective study	Regional Cancer Centre	Very low
Skeith 2017 [68] (Canada)	Thromboembolic conditions	N=162; mean age: 58.3 years	Cross-sectional study	Champlain Local Health Integration Network (Ontario, Canada)	Very low
Syrjala 2011 [69] (United States)	Cancer survivors treated with hemopoietic stem cell transplantation	N=775; mean age: 51.7 years	Randomized controlled trial	Fred Hutchinson Cancer Research Center (Seattle, Washington)	Moderate
Testa 2005 [70] (Italy)	Thromboembolic conditions	N=1393	Retrospective study	11 peripheral health care units and homes of 20 self-testing patients	Low
Woods 2000 [72] (United States)	Sickle cell disease	N=466; mean age: 27 years	Retrospective study	Telemedicine clinical sites of The Medical College of Georgia Sickle Cell Center (Augusta, Georgia)	Very low
Woods 1999 [73] (United States)	Sickle cell disease	N=120; standard (n=60), age: 33.32 years; telemedicine (n=60), age: 29.37 years	Randomized controlled trial	Telemedicine clinical sites of The Medical College of Georgia Sickle Cell Center (Augusta, Georgia)	Very low
Woods 1998 [71] (United States)	Sickle cell disease	N=28; mean age: 29.1 years	Pilot study	Remote telemedicine clinic site in Macon (affiliated with Medical Center of Central Georgia)	Very low
Wright 2007 [74] (Canada)	Allogenic blood and hemopoietic stem cell transplantation	N=1	Case study	Princess Margaret Hospital (Toronto, Ontario)	Very low

### Methodological Quality of the Studies

Of the 32 studies, 11 (34%) were pilot studies [42,51,62-65,71,75,76,78,80], 9 (28%) were retrospective studies [55-58,60,67,70,72,81], 6 (19%) were RCTs [52,53,61,69,73,77], 2 (6%) were cross-sectional studies [68,79], 1 (3%) was a case study [74], 1 (3%) was a pre-post study [54], 1 (3%) was a noncomparative prospective study [59], and 1 (3%) was a prospective cohort study [66]. Of the 6 RCTs, 1 (17%) was a single-blinded study and the remaining 5 (83%) had no blinding. Follow-up activity after the telemedicine interventions was tracked in six of the studies in the forms of postintervention neuroimaging examination (n=1, 17%) [77], follow-up assessments (n=3, 50%) [52,53,64], number of patient visits (n=1, 17%) [62], and interview (n=1, 17%) [82]. Based on the GRADE criteria, four included studies were rated as “moderate” [52,53,69,77], four were rated as “low” [54,61,70,79], and 24 were rated as “very low” [42,51,55-60,62-68,71-76,78,80,81]. The “low” and “very low” ratings were mainly a result of the type of study and indirectness of evidence.

### Description of Telemedicine Approaches

Table 3 and Table 4 provide detailed descriptions of the study purpose, telemedicine intervention used, and main findings of the included pediatric and adult studies, respectively. Additional study outcomes for pediatric and adult studies are also reported in Multimedia Appendix 2 and Multimedia Appendix 3, respectively. There were the following three main categories of telemedicine interventions in the included studies: video (n=9) [53,58,64,71-75,78], telephone (n=9) [52,55,59-61,65,67,76,81], and web (n=14) [42,51,54,56,57,62,63,66,68-70,77,79,80]. Video interventions used cameras and videoconferences to visually connect the patients to the providers. Telephone interventions involved nurse triages, counseling sessions over the phone, and telephone help lines available to patients 24/7. Web-based telemedicine interventions included online interfaces for patients to send and store data and to monitor their conditions from home while still maintaining intermittent contact with practitioners.

**Table 3:** Summary of the interventions and outcomes of the included studies in pediatric populations.

Source (country)	Purpose	Telemedicine intervention	Main findings
Adler 2015 [75] (Canada)	Improve outcomes and quality of life for children with cancer and blood disorders in the Caribbean by using telemedicine	Utilizing telemedicine facilities to conduct patient case consultations and discussions in real time	Satisfaction: Adequate to excellent overall attendee satisfaction rates Good to excellent patient care education satisfaction rates
Agarwal 2014 [76] (India)	Leverage an online collaborative information technology platform to improve outcomes of children with thalassemia receiving bone marrow transplants	Online open-access database with data storage, telemedicine, and knowledge exchange capabilities	Clinical outcomes: Comparable health outcomes with decreased cost Increased rates of family screening Targeted prenatal diagnoses for pregnant women
Cox 2015 [77] (United States)	Evaluate the feasibility and acceptability of a remote automated intervention to address late cognitive effects among childhood cancer survivors	Automated rotating exercises to train visual-spatial and verbal working memory over a 5- to 9-week period	Feasibility: Strong compliance with the intervention (88%) and pre- and postintervention imaging (91% and 93%, respectively) Satisfaction: Caregivers viewed the program as beneficial (70%) and would recommend it to others (93%)
Jacobson 2016 [78] (United States)	Evaluate the feasibility of using videoconferencing to assess breakthrough bleeds in children with severe hemophilia	Videoconferencing was utilized by health care providers to assess the patient’s condition during breakthrough bleeding	Satisfaction: Comparable or improved satisfaction was reported among health care providers and caregivers with videoconferencing versus phone call
Johnston 2017 [79] (Canada)	Analyze the use of e-consult by primary care providers for pediatric oncology and hematology conditions	Web portal based e-consult service for pediatric oncology and hematology	Satisfaction: High satisfaction ratings of e-consult services from primary care providers and pediatric hematology/oncology specialists Feasibility: 40% deferral of consults that were originally supposed to be in person
Pedrosa 2017 [80] (Brazil)	Evaluate the efficacy of a telemedicine-based knowledge-sharing program to improve management of childhood acute lymphoblastic leukemia	Teleconferencing-based knowledge-sharing program between hospitals in the United States and Brazil	Clinical outcomes: Decreases in overall mortality (31.0% to 12.8%), early death (8.1% to 3.6%), and relapse (10.2% to 7.9%) after institution of the telemedicine program
Stulac 2016 [81] (United States/Rwanda)	Evaluate the impact of partnership-based treatment of pediatric cancer patients in Rwanda by physicians and nurses based in Rwanda and United States	Collaboration between clinicians based in Rwanda and United States via telephone, email, and online databases to manage pediatric cancer cases	Clinical outcomes: Mean overall survival of 31 months Mean disease-free survival of 18 months

**Table 4:** Summary of the interventions and outcomes of the included studies in adult populations.

Source (Country)	Purpose	Telemedicine intervention	Main findings
Agrawal 2014 [51] (India)	Improve blood donor recruitment, retention, and relationship management	Call center staffed with personnel to conduct phone calls and send SMS text messages to recruit and build relationships with blood donors	Feasibility: Telerecruitment contributed to 63% of in-house and 13% of total donations Clinical outcomes: Telerecruitment helped establish relationships with blood donors and the society in general
Applebaum 2012 [52] (United States)	Determine the relationship between therapeutic alliance through telephone delivered cognitive behavioral therapy and psychotherapy outcomes in survivors of hemopoietic stem cell transplantation with posttraumatic stress disorder	Telephone-administered cognitive behavioral therapy	Clinical outcomes: Therapeutic alliance by telephone cognitive behavior therapy predicted decreased depressive symptoms, decreased general distress, and lower likelihood for re-experiencing symptoms
Bakitas 2015 [53] (United States)	Investigate the effect of early versus delayed palliative care on the quality of life of advanced-stage cancer patients	Weekly telehealth nurse coaching sessions	Clinical outcomes: No statistically significant evidence to support improved patient-reported outcomes in early versus late palliative care groups Statistically significant improved 1-year survival rate in early versus late palliative care groups (*P*=.04)
Barcellona 2013 [54] (Italy)	Compare the effect of a point-of-care home monitoring testing device supplemented by telemedicine with conventional monitoring in patients chronically treated with anti-vitamin K antagonists	TAONet telemedicine platform allowed patients to send international normalized ratio (INR) results and other clinical information to the Thrombosis Centre, as well as communicate with providers, adjust medications, and screen for serious events	Clinical outcomes: Greater blood checks and fewer missed INR checks in the home monitoring group Significant increase in time spent within the therapeutic range in the unstable group with home monitoring compared to conventional monitoring No significant difference in time spent within the therapeutic range in the stable group with home monitoring compared to conventional monitoring
Blissit 2015 [55] (United States)	Compare the effect of telephone versus face-to-face care on time spent within the therapeutic range for patients on warfarin	Pharmacist-managed care via telephone for patients taking warfarin	Clinical outcomes: No significant difference in time spent within the therapeutic range, significant bleeding rates, death rates, and thromboembolic events between face-to-face and telephone groups
Breen 2015 [42] (Australia)	Evaluate a real-time remote telemedicine system to improve monitoring and management of side-effects in patients with blood cancers	Smart phone app collected ambulatory patient health data in real time and transmitted this information to the treatment hospital where alerts were generated for actioning based on imputed patient data	Satisfaction: Patients reported increased feelings of empowerment and health awareness and adherence with the use of the application Clinical outcomes: Patients were better able to recall side-effects when using the application.
Burwick 2018 [56] (United States)	Identify ways to improve care of patients with monoclonal gammopathy of undetermined significance through e-consult use	Review of electronic hematology consults for monoclonal gammopathy of undetermined significance through e-consult use	Accessibility: Short time (2 days) to completion of e-consult Majority of e-consults were low risk
Cecchini 2016 [57] (United States)	Evaluate the efficacy of e-consults for the management of hematological disorders	Electronic consults with hematologists	Satisfaction: 65% of patients who responded said they preferred e-consults over face-to-face visits All providers who responded said they were either “satisfied” or “very satisfied” with e-consults Feasibility: 18% drop in face-to-face consults within 2 years of e-consult implementation
Clarke 2011 [58] (Canada)	Assess oncology telehealth usage in British Columbia	Oncology and genetic counselling appointments conducted with tele-conferencing units	Feasibility: Medical oncology teleconferences occurred more often than genetic counseling and medical genetics Clinical outcomes: A single medical oncologist conducted 58.7% of all telehealth encounters in 2009 Most common telehealth appointment types were gastrointestinal cancer and lymphoma
Compaci 2011 [59] (France)	Assess the feasibility and benefit of Ambulatory Medical Assistance phone calls in monitoring aggressive B-cell lymphoma treated with R-CHOP therapy	Standardized 10-minute telephone call twice a week by an oncology nurse to monitor vitals and side-effects during chemotherapy treatment	Clinical outcomes: Lower incidences of secondary hospitalization, delayed treatment, and reduced relative dose intensity, toxic death, and red blood cell transfusion compared to literature
Flannery 2009 [60] (United States)	Define telephone call volume, distribution, and reason in an ambulatory oncology practice	Telephone triage line managed by nurses to address patient symptoms	Feasibility: Seven calls were made or received for every 10 scheduled appointments Most calls were made on Monday mornings 30% of calls were made for more than one reason
Hung 2014 [61] (Australia)	Evaluate the impact of telephone counseling on nutrition and exercise after peripheral blood stem cell transplantation	Telephone counseling sessions conducted by a dietitian or exercise physiologist every 2 weeks for up to 100 days after transplantation	Clinical outcomes: Increased, but not statistically significant, protein intake (*P*=.17), cognitive functioning (*P*=.34), and social functioning (*P*=.17) in the extended care group versus usual care group Decreased, but not statistically significant, weight loss (*P*=.06) in the extended care group versus usual care group
Kirsh 2015 [62] (United States)	Analyze the impact of e-consults in improving specialty care access for veterans	E-consult service for various specialties	Feasibility: Hematology had the second highest (after cardiology) rate of e-consults Clinical outcomes: Within the first 3 months after an e-consult, there was a decreased likelihood of a subsequent face-to-face visit (*P*<.001) Within the first 3 months after an e-consult, there was an increased likelihood of a primary care visit (*P*<.001)
Najafi 2017 [63] (United States)	Evaluate the feasibility and acceptability (by providers) of an inpatient e-consult service	E-consult service for subspecialist consults	Feasibility: Majority of hospitalists and consultants believe that e-consults are easy to use and efficient Hematology had the second highest volume of e-consults Satisfaction: All hospitalists who completed the survey were satisfied with quality 43% of consultants who completed the survey felt they were able to provide high-quality care
Overend 2008 [64] (Canada)	Determine the efficacy, safety, and patient satisfaction of a nurse-led teleclinic to manage patients with indolent and chronic hematological malignancies	Nurse-led teleclinic interviews for hematology/oncology patients in between oncologist visits	Satisfaction: 82% of patients felt strongly that they could talk easily and openly, and that the provider was able to understand their situation to provide satisfactory care 62% reported they would participate in a teleclinic again Accessibility: Majority of patients did not feel that they needed to see a physician in person
Philip 2015 [65] (United States)	Evaluate the efficacy of a telephone-based pharmacist anticoagulation service	Telephone-based anticoagulation service run by pharmacists	Clinical outcomes: Increase in clinical pharmacy patient volume at ambulatory care clinics after the intervention No significant difference in time in the therapeutic range, hospitalization from thrombotic events or bleeding, work hours, and project completion rates between groups
Prochaska 2017 [66] (Germany)	Compare the outcomes of oral anticoagulation patients managed by an eHealth-based coagulation service versus regular medical care	Electronic file was used to manage medication and data at remote locations and interface between patients and providers	Clinical outcomes: eHealth service participants had lower rates of bleeding, hospitalization, and all-cause mortality compared to participants who received regular medical care
Reid 2011 [67] (Ireland)	Investigate the usage and patient/caller profile of a nurse-led chemotherapy telephone helpline	Telephone service run by nurses experienced in oncology and chemotherapy to address patient questions and concerns	Feasibility: 7498 calls received by helpline service 35.2% of patients called with multiple symptoms or concerns Accessibility: 47.5% of face-to-face consultations were avoided 4.3% of e-consults resulted in a follow-up referral that was not already scheduled Clinical outcomes: 36.8% of calls led to direct medical assessment
Skeith 2017 [68] (Canada)	Analyze the use and impact of e-consults in managing thromboembolic conditions	E-consult service for thrombosis medicine	Feasibility: Most common referral topics were thrombophilia testing, superficial venous thrombosis, and venous thromboembolism anticoagulation Satisfaction: Positive responses by primary care providers regarding the e-consult service
Syrjala 2011 [69] (United States)	Describe the development and feasibility of a new online survivorship care program for cancer patients treated with hemopoietic stem cell transplantation	Survivorship care delivered remotely through an online platform	Feasibility: 57% of participants required staff contact at least one time, usually for minor technical issues, or help in enrollment or the baseline assessment Most contacts were initiated by email instead of telephone
Testa 2005 [70] (Italy)	Implement a telemedicine system to deliver the same quality of care as traditional medicine in anticoagulation management, especially for those living far away from an anticoagulation center	Remote anticoagulation management via an electronic medical record system through which patients or health centers can send INR data to an anticoagulation clinic	Clinical outcomes: No difference in time in the therapeutic range between anticoagulation clinics (73%) and telemedicine use in general practitioner units (73.4%) Telemedicine use in nursing homes showed a lower percentage in the therapeutic range (66%) compared to anticoagulation clinics (73%) No difference in major complication rates between telemedicine use in peripheral units and anticoagulation clinics
Woods 2000 [72] (United States)	Evaluate the efficacy of the Georgia statewide telemedicine program in improving access to health services for patients with sick cell disease in remote areas	Telemedicine clinic for sickle cell patients with assistance of nurses at remote locations	Feasibility: Progressive increase in telemedicine clinic productivity over the course of the study Accessibility: Rural outreach increased from 19% to 29% of total clinic activity over the course of the study
Woods 1999 [73] (United States)	Compare patient satisfaction between telemedicine encounters and standard care for the management of sickle cell disease in adults	Telemedicine clinic for sickle cell patients with assistance of nurses at remote locations	Satisfaction: No significant difference in patient satisfaction between telemedicine and standard care groups Patients in the standard care group were more likely to offer positive open-ended comments than the telemedicine group Reasons for negative comments in the telemedicine group included confidentiality, technology, and access
Woods 1998 [71] (United States)	Evaluate the efficacy of a telemedicine clinic for adult sickle cell patients	Telemedicine clinic for sickle cell patients with assistance of nurses at remote locations	Clinical outcomes: No significant difference in clinic encounter time between telemedicine and standard care groups
Wright 2007 [74] (Canada)	Evaluate the efficacy and acceptability of an e-clinic for management of allogeneic blood and stem cell transplant patients	E-clinic visits at local health centers using videoconferencing technology	Clinical outcomes: High adherence rate for follow-up visits (87.5%) Improved symptom management Satisfaction: High satisfaction with e-clinic visits

### Study Outcomes in Video Telemedicine Interventions

#### Pediatric Video Interventions

Adler et al reported greater than 80% case review round attendee satisfaction with telemedicine case consultations and patient care education for children with cancer and blood disorders [75]. Improved satisfaction and communication among health care providers and caregivers with the use of videoconferencing at home for children with hemophilia were also noted by Jacobson et al [78].

#### Adult Video Interventions

Many telehealth nursing sessions have shown positive outcomes for patients with malignant hematological conditions [53]. Likewise, patients with hematological malignancies felt that they could talk easily and openly with the nurse who led telemedicine follow-up visits in between visits with the oncologist [64]. Telemedicine reportedly improved productivity [72] and increased mean cognitive skills quotient scores for patients [73], but caused no significant decrease in clinic encounter times [71]. Wright et al reported high follow-up adherence rates, good condition management, and high satisfaction for e-clinic visits in allogenic blood and stem cell transplant patients [74]. Clarke et al found that teleconsults in British Columbia were most often used with medical oncologists [58].

### Study Outcomes in Telephone Telemedicine Interventions

#### Pediatric Telephone Interventions

Agarwal et al found comparable health outcomes at a lower cost through the implementation of an online information technology database platform for patients receiving bone marrow transplants [76]. Stulac et al found that 13 of 24 Rwandan patients with pediatric cancer were in remission at the end of data collection following collaboration with United States–based physicians and nurses through telemedicine interventions [81].

#### Adult Telephone Interventions

Telephone therapy was utilized by Applebaum et al, which resulted in decreased depressive symptoms in hematopoietic stem cell transplant survivors with concurrent posttraumatic stress disorder [52]. For patients taking anticoagulants, Blissit et al noticed that telephone interventions led to significantly lower rates of bleeding in patients compared to face-to-face visits [55]. However, Philip et al did not see a significant difference in time within the therapeutic range, hospitalization, or bleeding with a telephone-based anticoagulation service [65]. Reid et al and Flannery et al utilized phone services to address patient questions and symptoms [60,67]. Reid et al noted that 36.8% of calls led to a medical assessment [67]; however, Flannery et al reported no notable improvements with the telephone triage [60]. Biweekly calls for monitoring vitals, side-effects [59], and telephone counseling [61] have all shown benefits for patients with malignant hematological conditions.

### Study Outcomes in Web-Based Telemedicine Interventions

#### Pediatric Web-Based Interventions

Cox et al found that patients, families, and caregivers had high rates of intervention participation and reported a positive experience with the introduction of a remote automated working memory intervention for childhood cancer survivors [77]. Telemedicine interventions also received high ratings from primary care providers and resulted in hematology consult deferral, according to Johnston et al [79]. Similarly, Pedrosa et al reported positive outcomes, with decreased rates of mortality, early death, and acute lymphoblastic leukemia relapse following implementation of a telemedicine program that allowed for knowledge sharing between high- and low-income countries [80].

#### Adult Web-Based Interventions

Based on a survey by Checchini et al, more than half of patients with nonmalignant hematological disorders preferred e-consults over face-to-face visits, and satisfaction was noted in all providers [57]. Breen et al found increased feelings of empowerment, health awareness, and medication adherence with the use of a telemedicine-based smartphone app that directly transmitted health data to the hospital for patients with blood cancers [42]. Najafi et al also noted that the majority of hospitalists and consultants found e-consult to be a feasible administration of subspecialty health services at a remotely-located hospital [63]. Primary care providers were satisfied with e-consult technology in a survey by Skeith et al, especially since 47.5% of in-person visits were avoided through the use of this telemedicine intervention [68]. Kirsh et al also reported a decreased number of face-to-face visits in patients using e-consult telemedicine interventions [62]. Additionally. Burwick et al saw that e-consults for hematology cases in patients with monoclonal gammopathy of undetermined significance led to a decrease in the time it took to complete the consultation [56]. In a study by Syrjala et al that included cancer patients treated with hematopoietic stem cell transplantation, over half of participating patients used an online platform and reported the need for staff contact one or more times per day, usually for minor assistance [69]. Barcellona et al reported an increased number of blood checks, fewer missed international normalized ratio checks in the home monitoring group, and a significant increase in the time within the therapeutic range for patients treated with vitamin K antagonists when using a smartphone app to monitor treatment [54]. Vitamin K antagonist users also had lower rates of bleeding, hospitalization, and all-cause mortality with the use of an online electronic file [66]. Electronic medical record implementation for remote anticoagulation management, however, showed no significant difference in the time within the therapeutic range or major complication rates in nursing homes [70]. Agrawal et al found that a call center allowed for increased recruitment of blood donors [51].

## Discussion

### Principal Findings

Overall, the vast majority of telemedicine interventions studied in this systematic review were shown to have a positive or neutral impact on patients, families, and health care providers. Telemedicine was found to be particularly useful for rural communities, patients in countries with less access to care, and patients with chronic conditions that require routine monitoring and communication with doctors [53,64,71-73,75,78,80,81]. Most studies showed improved or similar outcomes in groups that utilized telemedicine compared to those that did not. However, more research would be beneficial to determine telemedicine’s role in hematology and future implications for its use in the clinical setting. This evidence could also potentially increase patient satisfaction and patient-reported health outcomes.

### Telemedicine Interventions in Hematology: Expansion of Team-Based Medicine

In the realm of hematology, many new telemedicine interventions have emerged in recent years to expand the reach of specialty health care and improve patient outcomes [83,84]. Kulkarni et al reported that school-based telemedicine has provided access to multidisciplinary teams for the treatment of hemophilia and related bleeding disorders [84]. In patients who have undergone hematopoietic stem cell transplantation, therapy compliance and surveillance are seen to be good areas to implement telemedicine [83].

### Telemedicine Interventions in Hematology: Areas of Further Study

Future use of telemedicine in hematology and other subspecialties should focus on increasing the usage of videoconferencing with physicians from patient homes and making telemedicine more accessible to a wider range of patient populations [85]. This is especially the case for rural areas or regions where specialty health care and access to medical resources are limited [86]. The current COVID-19 pandemic has shown the value of maintaining and developing different ways of patient care, such as through the increased implementation of telemedicine in clinics and hospitals [87]. Various specialties should investigate ways in which they can best implement telemedicine interventions into their own field to improve access and care [86]. The next steps should include the wide use of triage systems that allow patients to be screened for telemedicine use eligibility [87].

### Telemedicine Interventions in Other Medical Specialties

In addition to the use of telemedicine in hematological conditions, it is widely used in various other medical specialties [88-93]. Meta-analyses on telemental health care have reported that applying telemedicine to psychotherapy not only improves patient satisfaction and access to care by circumventing the stigma of seeking in-person mental health care, but also offers a more financially prudent and flexible method of treatment [88]. Telemedicine has also been reported to contribute to shorter lengths of hospital stay and lower hospital mortality [90]. Multiple studies have shown it to be a cost-effective and accessible provision of mental health services [88]. Positive user outcomes with telehealth implementation in emergency rooms have indicated another area of potential use [91]. Similarly, telemedicine has been seen to help in the secondary prevention of stroke and in transient ischemia attack patients, but more research is needed to bolster evidence [93]. Cancer survivors generally had positive attitudes toward self-management and eHealth, but there was variation in the care needs depending on the type of cancer [92]. Telemonitoring is rarely used in prenatal care, but has potential in monitoring pregnant women who are at risk for preterm delivery [89]. These studies emphasize the positive experiences patients and providers have had with telemedicine in various fields of medicine, which further suggests that telemedicine is feasible, accessible, and beneficial. Many of the telemedicine interventions used in these other medical specialties can also be applied to the setting of hematology.

### Barriers to Telemedicine

Though studies indicate high reports of satisfaction from patients and providers in malignant and nonmalignant hematology [57,68,79,94], the incorporation of telemedicine in modern medicine still faces limitations and organizational barriers, including legal liability, cost and reimbursement, and confidentiality concerns [15]. Patient barriers, such as age, computer literacy, and education, also make telemedicine difficult to implement universally [15]. Additionally, technological barriers, including inadequate bandwidth, technically challenged staff, and licensing issues, can limit the scope of telemedicine [15]. There are also concerns regarding the availability of resources in languages other than English [82], lack of notable improvements with telephone triaging [60], efficiency of face-to-face visits [71], and quality of care delivered via e-consult [63]. Funding for both staff and equipment, privacy concerns, internet connection, and home recording all need to be addressed when considering the feasibility of telemedicine [15,95-97]. Since the cost-effectiveness of telemedicine varies across delivery settings [9], more research needs to be done to substantiate evidence of improvement in patient outcomes with the use of telemedicine. Furthermore, the ethics of telemedicine and digital health use in the clinical setting must be discussed. The ethical and legal concerns regarding the use of telemedicine have delayed the rapid widespread implementation of these technologies in multiple aspects of health care [98-100]. Specifically, data confidentiality, patient privacy, physician-patient relationships, and informed consent are all areas that need to be addressed [99,101-103]. Further work must be done to ensure that data management is conducted properly and the social implications or risks are communicated clearly to patients and physicians using telemedicine in practice [99,101-103]. However, despite its barriers, the ability of telemedicine to mediate many of the shortcomings associated with traditional face-to-face consultations still makes telemedicine particularly attractive to many fields, especially hematology.

### Strengths

Our systematic review has a number of strengths. First, we conducted our review following the recommendations for rigorous systematic reviews [49]. Second, we used a highly sensitive and specific search strategy guided by a librarian information specialist. There was no restriction on the country of study in order to minimize publication bias by identifying as many relevant studies as possible. Additional resources were searched, including published systematic reviews, clinical trial registries, and multiple electronic databases. Third, we employed no date restrictions on our search; no included articles were published prior to 1998. Therefore, the possibility that we missed earlier studies is very small. Finally, two authors completed the review process independently at all stages.

### Limitations

Some potential methodological limitations of our systematic review must be addressed. First, some relevant articles could have been missed in our literature search, despite our comprehensive search strategy in different databases. Second, articles included in the review were strictly peer-reviewed, which could cause publication bias from reporting only positive study results [104]. Third, the ranges of sample size and age, differences between malignant and nonmalignant conditions, and types of telemedicine interventions varied greatly among the studies. Lastly, the number of studies eligible for the review was relatively low; however, this may be a result of the specificity of hematological conditions and the limited number of available publications about the topic.

### Conclusions

Telemedicine is a recognized and cost-effective way of managing hematological conditions. Evidence from this systematic review suggests that telemedicine provides similar or improved health care compared to traditional face-to-face care. Videoconferencing, telephone-based services, and web-based services were also well received by patients, families, and health care providers in both pediatric and adult settings. However, due to the limited total number of articles and low quality of evidence of the included studies, further research must be done to determine the efficacy and plausibility of widespread implementation of telemedicine in hematology.

## Appendix

Multimedia Appendix 1 Literature search strategies across different databases.

Multimedia Appendix 2 Additional results of the included studies in pediatric populations.

Multimedia Appendix 3 Additional results of the included studies in adult populations.

## Abbreviations

GRADE: Grades of Recommendation, Assessment, Development, and Evaluation
PRISMA: Preferred Reporting Items for Systematic Reviews and Meta-Analyses
RCT: randomized controlled trial
