# Supplementary material for: Telemedicine in Malignant and Nonmalignant Hematology: Systematic Review of Pediatric and Adult Studies
Source: JMIR Mhealth Uhealth. 2021 Jul 8;9(7):e29619. doi: 10.2196/29619 (PMC8299344; doi:10.2196/29619)
Supplement: Multimedia Appendix 1 [file mhealth_v9i7e29619_app1.docx]

**Multimedia Appendix 1.** Literature search strategies across different databases. **PubMed**

**Hematology Terms**

("Hematologic Diseases"[Mesh] OR hematol*[tiab] OR "Leukemia"[Mesh] OR Leukemia[tiab] OR Anemia[tiab] OR Aplasia[tiab] OR Blood Coagulation Disorder*[tiab] OR Coagulation Protein Disorder*[tiab] OR “Disseminated Intravascular Coagulation”[tiab] or Ecchymosis[tiab] OR “Platelet Storage Pool Deficiency”[tiab] OR “Protein S Deficiency”[tiab] OR Purpura[tiab] OR Thrombocythemia[tiab] OR “Vitamin K Deficiency”[tiab] OR Blood Platelet Disorder*[tiab] OR “Bernard-Soulier Syndrome”[tiab] OR “Gray Platelet Syndrome”[tiab] OR “Platelet Storage Pool Deficiency”[tiab] OR Thrombasthenia[tiab] OR Thrombocytopenia[tiab] OR Thrombocytosis[tiab] OR von Willebrand Disease*[tiab] OR Blood Protein Disorder*[tiab] OR Agammaglobulinemia[tiab] OR “Antithrombin III Deficiency”[tiab] OR Dysgammaglobulinemia[tiab] OR Hypergammaglobulinemia[tiab] OR Hypoproteinemia[tiab] OR “Protein C Deficiency”[tiab] OR “Protein S Deficiency”[tiab] OR Bone Marrow Disease*[tiab] OR Bone Marrow Neoplasm*[tiab] OR Myelodysplastic-Myeloproliferative Disease*[tiab] OR Myelodysplastic Syndrome*[tiab] OR Myeloproliferative Disorder*[tiab] OR “Fetal Erythroblastosis”[tiab] OR “Hydrops Fetalis”[tiab] OR Kernicterus[tiab] OR Hematologic Neoplasm*[tiab] OR Hemoglobinopath*[tiab] OR “Sickle Cell Anemia”[tiab] OR “Hemoglobin C Disease”[tiab] OR Thalassemia[tiab] OR Hemorrhagic Disorder*[tiab] OR Afibrinogenemia[tiab] OR “Disseminated Intravascular Coagulation”[tiab] OR “Factor V Deficiency”[tiab] OR “Factor VII Deficiency”[tiab] OR “Factor X Deficiency”[tiab] OR “Factor XI Deficiency”[tiab] OR “Factor XII Deficiency”[tiab] OR “Factor XIII Deficiency”[tiab] OR Hemophilia[tiab] OR Hemostatic Disorder*[tiab] OR Hypoprothrombinemia*[tiab] OR “Waterhouse-Friderichsen Syndrome”[tiab] OR “Wiskott-Aldrich Syndrome”[tiab] OR Leukocyte Disorder*[tiab] OR Eosinophilia[tiab] OR “Infectious Mononucleosis”[tiab] OR Leukocytosis[tiab] OR Leukopenia[tiab] OR Leukostasis[tiab] OR “Pelger-Huet Anomaly”[tiab] OR “Phagocyte Bactericidal Dysfunction”[tiab] OR Methemoglobinemia[tiab] OR Pancytopenia[tiab] OR Polycythemia[tiab] OR “Hematologic Pregnancy Complications”[tiab] OR Preleukemia[tiab] OR Sulfhemoglobinemia[tiab] OR Thrombophilia[tiab] OR “Activated Protein C Resistance”[tiab] OR “Antithrombin III Deficiency”[tiab])

**Telehealth Terms**

(“Telemedicine”[mesh] OR Telemedicine[tiab] or telemental[tiab] or telehealth[tiab] or telepsychiatry[tiab] or telemonitoring[tiab] or telepathology[tiab] or teleradiology[tiab] or telerehabilitation[tiab] or “remote consultation”[tiab] or Tele-medicine[tiab] or tele-mental[tiab] or tele-health[tiab] or tele-psychiatry[tiab] or tele-monitoring[tiab] or tele-pathology[tiab] or tele-radiology[tiab] or tele-rehabilitation[tiab] or eHealth[tiab] OR e-health[tiab] OR “mobile health”[tiab] OR mHealth[tiab] OR m-health[tiab] OR "Electronic Mail"[Mesh] OR "Videoconferencing"[Mesh] OR “electronic mail”[tiab] OR e-mail[tiab] OR email[tiab] OR videoconferenc*[tiab] OR electronic consult*[tiab] or econsult*[tiab])

Limited to 1980 to present

**Embase**

**Hematology terms**

'hematologic disease'/exp OR 'hematologic disease' OR hematol*:ti,ab OR haematol*:ti,ab OR 'blood clotting disorder*':ti,ab OR 'blood dyscrasia':ti,ab OR 'blood group incompatibility':ti,ab OR 'blood transfusion reaction*':ti,ab OR 'bone marrow disease':ti,ab OR cytopenia:ti,ab OR 'erythrocyte disorder*':ti,ab OR 'hyperviscosity syndrome':ti,ab OR 'lymphatic system disease*':ti,ab OR 'blood disease':ti,ab OR 'blood disorder':ti,ab OR 'hematopathy':ti,ab OR 'hemic and lymphatic diseases':ti,ab OR hemopathy:ti,ab OR sulphaemoglobinaemia:ti,ab OR 'bleeding disorder*':ti,ab OR 'bleeding tendency':ti,ab OR 'blood clotting factor deficiency':ti,ab OR 'disseminated intravascular clotting':ti,ab OR hypercoagulability:ti,ab OR hypocoagulability:ti,ab OR 'newborn hemorrhagic disease':ti,ab OR 'thrombocyte disorder':ti,ab OR 'blood group abo incompatibility':ti,ab OR 'rhesus incompatibility':ti,ab OR 'rhesus isoimmunization':ti,ab OR dysglobulinemia:ti,ab OR dysproteinemia:ti,ab OR 'hyper ige syndrome':ti,ab OR 'hyperimmunoglobulinemia d':ti,ab OR 'immunoglobulin deficiency':ti,ab OR paraproteinemia:ti,ab OR 'schnitzler syndrome':ti,ab OR 'transfusion associated graft versus host disease':ti,ab OR 'transfusion related acute lung injury':ti,ab OR anemia:ti,ab OR 'bone marrow aplasia':ti,ab OR 'bone marrow depression':ti,ab OR 'bone marrow edema':ti,ab OR 'bone marrow hypoplasia':ti,ab OR 'bone marrow necrosis':ti,ab OR 'bone marrow suppression':ti,ab OR 'bone marrow toxicity':ti,ab OR 'febrile bone marrow aplasia':ti,ab OR 'myelodysplastic syndrome':ti,ab OR acanthocytosis:ti,ab OR dyserythropoiesis:ti,ab OR elliptocytosis:ti,ab OR erythroblastopenia:ti,ab OR erythroblastosis:ti,ab OR erythrocytosis:ti,ab OR erythroleukemia:ti,ab OR erythropenia:ti,ab OR 'erythropoietic protoporphyria':ti,ab OR 'hellp syndrome':ti,ab OR hemoglobinopathy:ti,ab OR hemolysis:ti,ab OR 'intravascular hemolysis':ti,ab OR megalocytosis:ti,ab OR microcytosis:ti,ab OR reticulocytopenia:ti,ab OR reticulocytosis:ti,ab OR spherocytosis:ti,ab OR stomatocytosis:ti,ab OR leukemia:ti,ab OR lymphoma:ti,ab OR 'malignant histiocytosis':ti,ab OR 'malignant plasmacytoma':ti,ab OR mastocytoma:ti,ab OR myeloma:ti,ab OR 'chronic granulomatous disease':ti,ab OR 'leukocyte adhesion deficiency':ti,ab OR lymphocytotoxicity:ti,ab OR 'may hegglin anomaly':ti,ab OR mononucleosis:ti,ab OR 'plasma cell dyscrasia':ti,ab OR 'intestine lymphangiectasia':ti,ab OR lymphadenopathy:ti,ab OR lymphangiectasis:ti,ab OR lymphangitis:ti,ab OR 'lymphatic malformation':ti,ab OR 'lymphatic system tumor':ti,ab OR lymphedema:ti,ab OR lymphocele:ti,ab OR 'lymphoid hyperplasia':ti,ab OR 'lymphoproliferative disease':ti,ab OR reticuloendotheliosis:ti,ab OR 'spleen disease':ti,ab OR 'thymus disease':ti,ab OR aplasia:ti,ab OR 'blood coagulation disorder*':ti,ab OR 'coagulation protein disorder*':ti,ab OR ecchymosis:ti,ab OR purpura:ti,ab OR thrombocythemia:ti,ab OR 'vitamin k deficiency':ti,ab OR 'blood platelet disorder*':ti,ab OR 'bernard-soulier syndrome':ti,ab OR 'gray platelet syndrome':ti,ab OR 'platelet storage pool deficiency':ti,ab OR thrombasthenia:ti,ab OR thrombocytopenia:ti,ab OR thrombocytosis:ti,ab OR 'von willebrand disease*':ti,ab OR 'blood protein disorder*':ti,ab OR agammaglobulinemia:ti,ab OR dysgammaglobulinemia:ti,ab OR hypergammaglobulinemia:ti,ab OR hypoproteinemia:ti,ab OR 'protein c deficiency':ti,ab OR 'protein s deficiency':ti,ab OR 'bone marrow disease*':ti,ab OR 'bone marrow neoplasm*':ti,ab OR 'myelodysplastic-myeloproliferative disease*':ti,ab OR 'myelodysplastic syndrome*':ti,ab OR 'myeloproliferative disorder*':ti,ab OR 'fetal erythroblastosis':ti,ab OR 'hydrops fetalis':ti,ab OR kernicterus:ti,ab OR 'hematologic neoplasm*':ti,ab OR hemoglobinopath*:ti,ab OR 'sickle cell anemia':ti,ab OR 'hemoglobin c disease':ti,ab OR thalassemia:ti,ab OR 'hemorrhagic disorder*':ti,ab OR afibrinogenemia:ti,ab OR 'disseminated intravascular coagulation':ti,ab OR 'factor v deficiency':ti,ab OR 'factor vii deficiency':ti,ab OR 'factor x deficiency':ti,ab OR 'factor xi deficiency':ti,ab OR 'factor xii deficiency':ti,ab OR 'factor xiii deficiency':ti,ab OR hemophilia:ti,ab OR 'hemostatic disorder*':ti,ab OR hypoprothrombinemia*:ti,ab OR 'waterhouse-friderichsen syndrome':ti,ab OR 'wiskott-aldrich syndrome':ti,ab OR 'leukocyte disorder*':ti,ab OR eosinophilia:ti,ab OR 'infectious mononucleosis':ti,ab OR leukocytosis:ti,ab OR leukopenia:ti,ab OR leukostasis:ti,ab OR 'pelger-huet anomaly':ti,ab OR 'phagocyte bactericidal dysfunction':ti,ab OR methemoglobinemia:ti,ab OR pancytopenia:ti,ab OR polycythemia:ti,ab OR 'hematologic pregnancy complications':ti,ab OR preleukemia:ti,ab OR sulfhemoglobinemia:ti,ab OR thrombophilia:ti,ab OR 'activated protein c resistance':ti,ab OR 'antithrombin iii deficiency':ti,ab

**Telemedicine terms**

'telemedicine'/exp OR 'telehealth'/exp OR telemedicine:ti,ab OR telehealth:ti,ab OR telecardiology:ti,ab OR teleconsultation:ti,ab OR telediagnosis:ti,ab OR telemonitoring:ti,ab OR telepathology:ti,ab OR telepsychiatry:ti,ab OR teleradiology:ti,ab OR teleradiotherapy:ti,ab OR telerehabilitation:ti,ab OR telesurgery:ti,ab OR 'tele-medicine':ti,ab OR 'tele-cardiology':ti,ab OR 'tele-consultation':ti,ab OR 'tele-diagnosis':ti,ab OR 'tele-monitoring':ti,ab OR 'tele-pathology':ti,ab OR 'tele-psychiatry':ti,ab OR 'tele-radiology':ti,ab OR 'tele-radiotherapy':ti,ab OR 'tele-rehabilitation':ti,ab OR 'tele-surgery':ti,ab OR 'tele-health':ti,ab OR 'videoconferencing'/exp OR 'e-mail'/exp OR ehealth:ti,ab OR 'mobile health':ti,ab OR mhealth:ti,ab OR 'm-health':ti,ab OR videoconferenc*:ti,ab OR 'electronic consult*':ti,ab OR econsult*:ti,ab

Limited to Articles, Articles in press, reviews, 1980-present

­­­­­­­­­­­­­­­­­­­­­

CENTRAL

Search Name: Sherif_telemedicine_hematology

Date Run: 06/02/18 22:40:12.801

Description:

ID Search Hits

#1 MeSH descriptor: [Hematologic Diseases] explode all trees 11511

#2 MeSH descriptor: [Leukemia] explode all trees 3682

#3 hematol* or Leukemia or Anemia or Aplasia or Blood Coagulation Disorder* or Coagulation Protein Disorder* or "Disseminated Intravascular Coagulation" or Ecchymosis or "Platelet Storage Pool Deficiency" or "Protein S Deficiency" or Purpura or Thrombocythemia or "Vitamin K Deficiency" or Blood Platelet Disorder* or "Bernard-Soulier Syndrome" or "Gray Platelet Syndrome" or "Platelet Storage Pool Deficiency" or Thrombasthenia or Thrombocytopenia or Thrombocytosis or von Willebrand Disease* or Blood Protein Disorder* or Agammaglobulinemia or "Antithrombin III Deficiency" or Dysgammaglobulinemia or Hypergammaglobulinemia or Hypoproteinemia or "Protein C Deficiency" or "Protein S Deficiency" or Bone Marrow Disease* or Bone Marrow Neoplasm* or Myelodysplastic-Myeloproliferative Disease* or Myelodysplastic Syndrome* or Myeloproliferative Disorder* or "Fetal Erythroblastosis" or "Hydrops Fetalis" or Kernicterus or Hematologic Neoplasm* or Hemoglobinopath* or "Sickle Cell Anemia" or "Hemoglobin C Disease" or Thalassemia or Hemorrhagic Disorder* or Afibrinogenemia or "Disseminated Intravascular Coagulation" or "Factor V Deficiency" or "Factor VII Deficiency" or "Factor X Deficiency" or "Factor XI Deficiency" or "Factor XII Deficiency" or "Factor XIII Deficiency" or Hemophilia or Hemostatic Disorder* or Hypoprothrombinemia* or "Waterhouse-Friderichsen Syndrome" or "Wiskott-Aldrich Syndrome" or Leukocyte Disorder* or Eosinophilia or "Infectious Mononucleosis" or Leukocytosis or Leukopenia or Leukostasis or "Pelger-Huet Anomaly" or "Phagocyte Bactericidal Dysfunction" or Methemoglobinemia or Pancytopenia or Polycythemia or "Hematologic Pregnancy Complications" or Preleukemia or Sulfhemoglobinemia or Thrombophilia or "Activated Protein C Resistance" or "Antithrombin III Deficiency" or hematol* or haematol* or "blood clotting disorder*" or "blood dyscrasia" or "blood group incompatibility" or "blood protein disorder*" or "blood transfusion reaction*" or "bone marrow disease" or cytopenia or "erythrocyte disorder*" or "hyperviscosity syndrome" or "leukocyte disorder*" or "lymphatic system disease*" or "blood disease" or "blood disorder" or "hematopathy" or "hemic and lymphatic diseases" or hemopathy or sulfhemoglobinemia or sulphaemoglobinaemia or "activated protein C resistance" or "bleeding disorder*" or "bleeding tendency" or "blood clotting factor deficiency" or "disseminated intravascular clotting" or hypercoagulability or hypocoagulability or "newborn hemorrhagic disease" or "thrombocyte disorder" or thromboembolism or "blood group ABO incompatibility" or "rhesus incompatibility" or "rhesus isoimmunization" or dysglobulinemia or dysproteinemia or "hyper IgE syndrome" or hypergammaglobulinemia or "hyperimmunoglobulinemia D" or hypoproteinemia or "immunoglobulin deficiency" or paraproteinemia or "protein C deficiency" or "protein S deficiency" or "Schnitzler syndrome" or "transfusion associated graft versus host disease" or "transfusion related acute lung injury" or anemia or "bone marrow aplasia" or "bone marrow depression" or "bone marrow edema" or "bone marrow hypoplasia" or "bone marrow necrosis" or "bone marrow suppression" or "bone marrow toxicity" or dyserythropoiesis or erythropenia or "febrile bone marrow aplasia" or leukopenia or "myelodysplastic syndrome" or "myeloproliferative disorder*" or reticulocytopenia or acanthocytosis or dyserythropoiesis or elliptocytosis or erythroblastopenia or erythroblastosis or erythrocytosis or erythroleukemia or erythropenia or "erythropoietic protoporphyria" or "HELLP syndrome" or hemoglobinopathy or hemolysis or "intravascular hemolysis" or megalocytosis or methemoglobinemia or microcytosis or reticulocytopenia or reticulocytosis or spherocytosis or stomatocytosis or leukemia or lymphoma or "malignant histiocytosis" or "malignant plasmacytoma" or mastocytoma or myeloma or "chronic granulomatous disease" or "leukocyte adhesion deficiency" or leukocytosis or leukopenia or leukostasis or lymphocytotoxicity or "May Hegglin anomaly" or mononucleosis or "plasma cell dyscrasia" or "intestine lymphangiectasia" or lymphadenopathy or lymphangiectasis or lymphangitis or "lymphatic malformation" or "lymphatic system tumor" or lymphedema or lymphocele or "lymphoid hyperplasia" or "lymphoproliferative disease" or reticuloendotheliosis or "spleen disease" or "thymus disease" or Aplasia or "Blood Coagulation Disorder*" or "Coagulation Protein Disorder*" or "Disseminated Intravascular Coagulation" or Ecchymosis or "Platelet Storage Pool Deficiency" or "Protein S Deficiency" or Purpura or Thrombocythemia or "Vitamin K Deficiency" or "Blood Platelet Disorder*" or "Bernard-Soulier Syndrome" or "Gray Platelet Syndrome" or "Platelet Storage Pool Deficiency" or Thrombasthenia or Thrombocytopenia or Thrombocytosis or "von Willebrand Disease*" or "Blood Protein Disorder*" or Agammaglobulinemia or "Antithrombin III Deficiency" or Dysgammaglobulinemia or Hypergammaglobulinemia or Hypoproteinemia or "Protein C Deficiency" or "Protein S Deficiency" or "Bone Marrow Disease*" or "Bone Marrow Neoplasm*" or "Myelodysplastic-Myeloproliferative Disease*" or "Myelodysplastic Syndrome*" or "Myeloproliferative Disorder*" or "Fetal Erythroblastosis" or "Hydrops Fetalis" or Kernicterus or "Hematologic Neoplasm*" or Hemoglobinopath* or "Sickle Cell Anemia" or "Hemoglobin C Disease" or Thalassemia or "Hemorrhagic Disorder*" or Afibrinogenemia or "Disseminated Intravascular Coagulation" or "Factor V Deficiency" or "Factor VII Deficiency" or "Factor X Deficiency" or "Factor XI Deficiency" or "Factor XII Deficiency" or "Factor XIII Deficiency" or Hemophilia or "Hemostatic Disorder*" or Hypoprothrombinemia* or "Waterhouse-Friderichsen Syndrome" or "Wiskott-Aldrich Syndrome" or "Leukocyte Disorder*" or Eosinophilia or "Infectious Mononucleosis" or Leukocytosis or Leukopenia or Leukostasis or "Pelger-Huet Anomaly" or "Phagocyte Bactericidal Dysfunction" or Methemoglobinemia or Pancytopenia or Polycythemia or "Hematologic Pregnancy Complications" or Preleukemia or Sulfhemoglobinemia or Thrombophilia or "Activated Protein C Resistance" or "Antithrombin III Deficiency" 72340

#4 #1 or #2 or #3 73325

#5 MeSH descriptor: [Telemedicine] explode all trees 2146

#6 MeSH descriptor: [Electronic Mail] explode all trees 294

#7 MeSH descriptor: [Videoconferencing] explode all trees 173

#8 telemedicine or telehealth or telecardiology or teleconsultation or teledermatology or telediagnosis or telemonitoring or telepathology or telepsychiatry or teleradiology or teleradiotherapy or telerehabilitation or telesurgery or teletherapy or tele-medicine or tele-cardiology or tele-consultation or tele-dermatology or tele-diagnosis or tele-monitoring or tele-pathology or tele-psychiatry or tele-radiology or tele-radiotherapy or tele-rehabilitation or tele-surgery or tele-therapy or tele-health or eHealth or e-health or "mobile health" or mHealth or m-health or videoconferenc* or "electronic consult*" or econsult* 5421

#9 #5 or #6 or #7 or #8 5875

#10 #4 and #9 170

**CENTRAL = 86**

**CINAHL**

( (MH "Hematologic Diseases+") OR (MH "Leukemia+") ) OR TI ( hematol* or Leukemia or Anemia or Aplasia or Blood Coagulation Disorder* or Coagulation Protein Disorder* or "Disseminated Intravascular Coagulation" or Ecchymosis or "Platelet Storage Pool Deficiency" or "Protein S Deficiency" or Purpura or Thrombocythemia or "Vitamin K Deficiency" or Blood Platelet Disorder* or "Bernard-Soulier Syndrome" or "Gray Platelet Syndrome" or "Platelet Storage Pool Deficiency" or Thrombasthenia or Thrombocytopenia or Thrombocytosis or von Willebrand Disease* or Blood Protein Disorder* or Agammaglobulinemia or "Antithrombin III Deficiency" or Dysgammaglobulinemia or Hypergammaglobulinemia or Hypoproteinemia or "Protein C Deficiency" or "Protein S Deficiency" or Bone Marrow Disease* or Bone Marrow Neoplasm* or Myelodysplastic-Myeloproliferative Disease* or Myelodysplastic Syndrome* or Myeloproliferative Disorder* or "Fetal Erythroblastosis" or "Hydrops Fetalis" or Kernicterus or Hematologic Neoplasm* or Hemoglobinopath* or "Sickle Cell Anemia" or "Hemoglobin C Disease" or Thalassemia or Hemorrhagic Disorder* or Afibrinogenemia or "Disseminated Intravascular Coagulation" or "Factor V Deficiency" or "Factor VII Deficiency" or "Factor X Deficiency" or "Factor XI Deficiency" or "Factor XII Deficiency" or "Factor XIII Deficiency" or Hemophilia or Hemostatic Disorder* or Hypoprothrombinemia* or "Waterhouse-Friderichsen Syndrome" or "Wiskott-Aldrich Syndrome" or Leukocyte Disorder* or Eosinophilia or "Infectious Mononucleosis" or Leukocytosis or Leukopenia or Leukostasis or "Pelger-Huet Anomaly" or "Phagocyte Bactericidal Dysfunction" or Methemoglobinemia or Pancytopenia or Polycythemia or "Hematologic Pregnancy Complications" or Preleukemia or Sulfhemoglobinemia or Thrombophilia or "Activated Protein C Resistance" or "Antithrombin III Deficiency" or hematol* or haematol* or "blood clotting disorder*" or "blood dyscrasia" or "blood group incompatibility" or "blood protein disorder*" or "blood transfusion reaction*" or "bone marrow disease" or cytopenia or "erythrocyte disorder*" or "hyperviscosity syndrome" or "leukocyte disorder*" or "lymphatic system disease*" or "blood disease" or "blood disorder" or "hematopathy" or "hemic and lymphatic diseases" or hemopathy or sulfhemoglobinemia or sulphaemoglobinaemia or "activated protein C resistance" or "bleeding disorder*" or "bleeding tendency" or "blood clotting factor deficiency" or "disseminated intravascular clotting" or hypercoagulability or hypocoagulability or "newborn hemorrhagic disease" or "thrombocyte disorder" or thromboembolism or "blood group ABO incompatibility" or "rhesus incompatibility" or "rhesus isoimmunization" or dysglobulinemia or dysproteinemia or "hyper IgE syndrome" or hypergammaglobulinemia or "hyperimmunoglobulinemia D" or hypoproteinemia or "immunoglobulin deficiency" or paraproteinemia or "protein C deficiency" or "protein S deficiency" or "Schnitzler syndrome" or "transfusion associated graft versus host disease" or "transfusion related acute lung injury" or anemia or "bone marrow aplasia" or "bone marrow depression" or "bone marrow edema" or "bone marrow hypoplasia" or "bone marrow necrosis" or "bone marrow suppression" or "bone marrow toxicity" or dyserythropoiesis or erythropenia or "febrile bone marrow aplasia" or leukopenia or "myelodysplastic syndrome" or "myeloproliferative disorder*" or reticulocytopenia or acanthocytosis or dyserythropoiesis or elliptocytosis or erythroblastopenia or erythroblastosis or erythrocytosis or erythroleukemia or erythropenia or "erythropoietic protoporphyria" or "HELLP syndrome" or hemoglobinopathy or hemolysis or "intravascular hemolysis" or megalocytosis or methemoglobinemia or microcytosis or reticulocytopenia or reticulocytosis or spherocytosis or stomatocytosis or leukemia or lymphoma or "malignant histiocytosis" or "malignant plasmacytoma" or mastocytoma or myeloma or "chronic granulomatous disease" or "leukocyte adhesion deficiency" or leukocytosis or leukopenia or leukostasis or lymphocytotoxicity or "May Hegglin anomaly" or mononucleosis or "plasma cell dyscrasia" or "intestine lymphangiectasia" or lymphadenopathy or lymphangiectasis or lymphangitis or "lymphatic malformation" or "lymphatic system tumor" or lymphedema or lymphocele or "lymphoid hyperplasia" or "lymphoproliferative disease" or reticuloendotheliosis or "spleen disease" or "thymus disease" or Aplasia or "Blood Coagulation Disorder*" or "Coagulation Protein Disorder*" or "Disseminated Intravascular Coagulation" or Ecchymosis or "Platelet Storage Pool Deficiency" or "Protein S Deficiency" or Purpura or Thrombocythemia or "Vitamin K Deficiency" or "Blood Platelet Disorder*" or "Bernard-Soulier Syndrome" or "Gray Platelet Syndrome" or "Platelet Storage Pool Deficiency" or Thrombasthenia or Thrombocytopenia or Thrombocytosis or "von Willebrand Disease*" or "Blood Protein Disorder*" or Agammaglobulinemia or "Antithrombin III Deficiency" or Dysgammaglobulinemia or Hypergammaglobulinemia or Hypoproteinemia or "Protein C Deficiency" or "Protein S Deficiency" or "Bone Marrow Disease*" or "Bone Marrow Neoplasm*" or "Myelodysplastic-Myeloproliferative Disease*" or "Myelodysplastic Syndrome*" or "Myeloproliferative Disorder*" or "Fetal Erythroblastosis" or "Hydrops Fetalis" or Kernicterus or "Hematologic Neoplasm*" or Hemoglobinopath* or "Sickle Cell Anemia" or "Hemoglobin C Disease" or Thalassemia or "Hemorrhagic Disorder*" or Afibrinogenemia or "Disseminated Intravascular Coagulation" or "Factor V Deficiency" or "Factor VII Deficiency" or "Factor X Deficiency" or "Factor XI Deficiency" or "Factor XII Deficiency" or "Factor XIII Deficiency" or Hemophilia or "Hemostatic Disorder*" or Hypoprothrombinemia* or "Waterhouse-Friderichsen Syndrome" or "Wiskott-Aldrich Syndrome" or "Leukocyte Disorder*" or Eosinophilia or "Infectious Mononucleosis" or Leukocytosis or Leukopenia or Leukostasis or "Pelger-Huet Anomaly" or "Phagocyte Bactericidal Dysfunction" or Methemoglobinemia or Pancytopenia or Polycythemia or "Hematologic Pregnancy Complications" or Preleukemia or Sulfhemoglobinemia or Thrombophilia or "Activated Protein C Resistance" or "Antithrombin III Deficiency" ) OR AB ( hematol* or Leukemia or Anemia or Aplasia or Blood Coagulation Disorder* or Coagulation Protein Disorder* or "Disseminated Intravascular Coagulation" or Ecchymosis or "Platelet Storage Pool Deficiency" or "Protein S Deficiency" or Purpura or Thrombocythemia or "Vitamin K Deficiency" or Blood Platelet Disorder* or "Bernard-Soulier Syndrome" or "Gray Platelet Syndrome" or "Platelet Storage Pool Deficiency" or Thrombasthenia or Thrombocytopenia or Thrombocytosis or von Willebrand Disease* or Blood Protein Disorder* or Agammaglobulinemia or "Antithrombin III Deficiency" or Dysgammaglobulinemia or Hypergammaglobulinemia or Hypoproteinemia or "Protein C Deficiency" or "Protein S Deficiency" or Bone Marrow Disease* or Bone Marrow Neoplasm* or Myelodysplastic-Myeloproliferative Disease* or Myelodysplastic Syndrome* or Myeloproliferative Disorder* or "Fetal Erythroblastosis" or "Hydrops Fetalis" or Kernicterus or Hematologic Neoplasm* or Hemoglobinopath* or "Sickle Cell Anemia" or "Hemoglobin C Disease" or Thalassemia or Hemorrhagic Disorder* or Afibrinogenemia or "Disseminated Intravascular Coagulation" or "Factor V Deficiency" or "Factor VII Deficiency" or "Factor X Deficiency" or "Factor XI Deficiency" or "Factor XII Deficiency" or "Factor XIII Deficiency" or Hemophilia or Hemostatic Disorder* or Hypoprothrombinemia* or "Waterhouse-Friderichsen Syndrome" or "Wiskott-Aldrich Syndrome" or Leukocyte Disorder* or Eosinophilia or "Infectious Mononucleosis" or Leukocytosis or Leukopenia or Leukostasis or "Pelger-Huet Anomaly" or "Phagocyte Bactericidal Dysfunction" or Methemoglobinemia or Pancytopenia or Polycythemia or "Hematologic Pregnancy Complications" or Preleukemia or Sulfhemoglobinemia or Thrombophilia or "Activated Protein C Resistance" or "Antithrombin III Deficiency" or hematol* or haematol* or "blood clotting disorder*" or "blood dyscrasia" or "blood group incompatibility" or "blood protein disorder*" or "blood transfusion reaction*" or "bone marrow disease" or cytopenia or "erythrocyte disorder*" or "hyperviscosity syndrome" or "leukocyte disorder*" or "lymphatic system disease*" or "blood disease" or "blood disorder" or "hematopathy" or "hemic and lymphatic diseases" or hemopathy or sulfhemoglobinemia or sulphaemoglobinaemia or "activated protein C resistance" or "bleeding disorder*" or "bleeding tendency" or "blood clotting factor deficiency" or "disseminated intravascular clotting" or hypercoagulability or hypocoagulability or "newborn hemorrhagic disease" or "thrombocyte disorder" or thromboembolism or "blood group ABO incompatibility" or "rhesus incompatibility" or "rhesus isoimmunization" or dysglobulinemia or dysproteinemia or "hyper IgE syndrome" or hypergammaglobulinemia or "hyperimmunoglobulinemia D" or hypoproteinemia or "immunoglobulin deficiency" or paraproteinemia or "protein C deficiency" or "protein S deficiency" or "Schnitzler syndrome" or "transfusion associated graft versus host disease" or "transfusion related acute lung injury" or anemia or "bone marrow aplasia" or "bone marrow depression" or "bone marrow edema" or "bone marrow hypoplasia" or "bone marrow necrosis" or "bone marrow suppression" or "bone marrow toxicity" or dyserythropoiesis or erythropenia or "febrile bone marrow aplasia" or leukopenia or "myelodysplastic syndrome" or "myeloproliferative disorder*" or reticulocytopenia or acanthocytosis or dyserythropoiesis or elliptocytosis or erythroblastopenia or erythroblastosis or erythrocytosis or erythroleukemia or erythropenia or "erythropoietic protoporphyria" or "HELLP syndrome" or hemoglobinopathy or hemolysis or "intravascular hemolysis" or megalocytosis or methemoglobinemia or microcytosis or reticulocytopenia or reticulocytosis or spherocytosis or stomatocytosis or leukemia or lymphoma or "malignant histiocytosis" or "malignant plasmacytoma" or mastocytoma or myeloma or "chronic granulomatous disease" or "leukocyte adhesion deficiency" or leukocytosis or leukopenia or leukostasis or lymphocytotoxicity or "May Hegglin anomaly" or mononucleosis or "plasma cell dyscrasia" or "intestine lymphangiectasia" or lymphadenopathy or lymphangiectasis or lymphangitis or "lymphatic malformation" or "lymphatic system tumor" or lymphedema or lymphocele or "lymphoid hyperplasia" or "lymphoproliferative disease" or reticuloendotheliosis or "spleen disease" or "thymus disease" or Aplasia or "Blood Coagulation Disorder*" or "Coagulation Protein Disorder*" or "Disseminated Intravascular Coagulation" or Ecchymosis or "Platelet Storage Pool Deficiency" or "Protein S Deficiency" or Purpura or Thrombocythemia or "Vitamin K Deficiency" or "Blood Platelet Disorder*" or "Bernard-Soulier Syndrome" or "Gray Platelet Syndrome" or "Platelet Storage Pool Deficiency" or Thrombasthenia or Thrombocytopenia or Thrombocytosis or "von Willebrand Disease*" or "Blood Protein Disorder*" or Agammaglobulinemia or "Antithrombin III Deficiency" or Dysgammaglobulinemia or Hypergammaglobulinemia or Hypoproteinemia or "Protein C Deficiency" or "Protein S Deficiency" or "Bone Marrow Disease*" or "Bone Marrow Neoplasm*" or "Myelodysplastic-Myeloproliferative Disease*" or "Myelodysplastic Syndrome*" or "Myeloproliferative Disorder*" or "Fetal Erythroblastosis" or "Hydrops Fetalis" or Kernicterus or "Hematologic Neoplasm*" or Hemoglobinopath* or "Sickle Cell Anemia" or "Hemoglobin C Disease" or Thalassemia or "Hemorrhagic Disorder*" or Afibrinogenemia or "Disseminated Intravascular Coagulation" or "Factor V Deficiency" or "Factor VII Deficiency" or "Factor X Deficiency" or "Factor XI Deficiency" or "Factor XII Deficiency" or "Factor XIII Deficiency" or Hemophilia or "Hemostatic Disorder*" or Hypoprothrombinemia* or "Waterhouse-Friderichsen Syndrome" or "Wiskott-Aldrich Syndrome" or "Leukocyte Disorder*" or Eosinophilia or "Infectious Mononucleosis" or Leukocytosis or Leukopenia or Leukostasis or "Pelger-Huet Anomaly" or "Phagocyte Bactericidal Dysfunction" or Methemoglobinemia or Pancytopenia or Polycythemia or "Hematologic Pregnancy Complications" or Preleukemia or Sulfhemoglobinemia or Thrombophilia or "Activated Protein C Resistance" or "Antithrombin III Deficiency" )

AND

( (MH "Telehealth+") OR (MH "Videoconferencing+") OR (MH "Electronic Mail") ) OR TI ( telemedicine or telehealth or telecardiology or teleconsultation or teledermatology or telediagnosis or telemonitoring or telepathology or telepsychiatry or teleradiology or teleradiotherapy or telerehabilitation or telesurgery or teletherapy or tele-medicine or tele-cardiology or tele-consultation or tele-dermatology or tele-diagnosis or tele-monitoring or tele-pathology or tele-psychiatry or tele-radiology or tele-radiotherapy or tele-rehabilitation or tele-surgery or tele-therapy or tele-health or eHealth or e-health or "mobile health" or mHealth or m-health or videoconferenc* or "electronic consult*" or econsult* ) OR AB ( telemedicine or telehealth or telecardiology or teleconsultation or teledermatology or telediagnosis or telemonitoring or telepathology or telepsychiatry or teleradiology or teleradiotherapy or telerehabilitation or telesurgery or teletherapy or tele-medicine or tele-cardiology or tele-consultation or tele-dermatology or tele-diagnosis or tele-monitoring or tele-pathology or tele-psychiatry or tele-radiology or tele-radiotherapy or tele-rehabilitation or tele-surgery or tele-therapy or tele-health or eHealth or e-health or "mobile health" or mHealth or m-health or videoconferenc* or "electronic consult*" or econsult* )

**Search results (Feb 7, 2018)**

PubMed: 226
Embase: 629
CENTRAL: 86
CINAHL: 39

Total: 980
Post deduplication: 811 🡪 sent in EndNote library 2/7/18
