# Supplementary material for: Telemedicine in Malignant and Nonmalignant Hematology: Systematic Review of Pediatric and Adult Studies
Source: JMIR Mhealth Uhealth. 2021 Jul 8;9(7):e29619. doi: 10.2196/29619 (PMC8299344; doi:10.2196/29619)
Supplement: Multimedia Appendix 2 [file mhealth_v9i7e29619_app2.docx]

**Multimedia Appendix 2.** Additional results of the included studies in pediatric populations.

| **Source (Country)** | **Additional results** |
| --- | --- |
| Adler 2015 (Canada) | - 54 cases reviewed, 35 presented - 4.07/5 overall satisfaction rating of case consultation review rounds - 4.18/5 overall satisfaction of patient care education rounds |
| Agarwal 2014 (India) | - 2,700 extended family members offered cascade screening and counseling - 2,400 patients registered and 112 bone marrow transplantations performed - 50 pregnant mothers offered targeted prenatal diagnosis |
| Cox 2015 (USA) | - 30 of the 34 participants completed the intervention - 91% of patients completed pre-intervention neuroimaging exams - 93% of patients completed post-intervention neuroimaging exams - Families had necessary skills to utilize the computer program successfully - 63% of caregivers were able to find time to complete training - 70% of caregivers viewed training as beneficial - 93% of caregivers would recommend this intervention to others |
| Jacobson 2016 (USA) | - 4 video visits completed with total of 3 patients - Video quality was fairly good, although mobile webcam quality was reportedly better - Healthcare providers and caregivers reported comparable or improved satisfaction regarding videoconferencing versus phone call |
| Johnston 2017 (Canada) | - 524 consults were seen in the pediatric hematology/oncology clinic - 90.5% of eConsults for hematology - 9.5% of eConsults for oncology - Common topics were hemoglobinopathy, anemia, bleeding disorder, and thrombotic state - Primary care providers rated eConsult service very highly and gave positive feedback - eConsult service resulted in deferral of 40% of consults originally thought to require face-to-face specialist visit - All eConsults took under 15 minutes to complete |
| Pedrosa 2017 (Brazil) | - 163 new patients were discussed during the study - Each patient was discussed 1.9 times on average - Compared with the era predating online meetings (1993–2005), overall mortality, early death, and relapse of patients with acute lymphoblastic leukemia decreased after the telemedicine program was instituted |
| Stulac 2016 (USA/Rwanda) | - Mean overall and disease-free survivals were 31 and 18 months, respectively - 15/24 patients received all chemotherapy doses as scheduled - Thirteen patients were in remission at the completion of data collection - Two patients died from treatment complications - Nine patients had progressive disease - No patients abandoned treatment |
