# Supplementary material for: Telemedicine in Malignant and Nonmalignant Hematology: Systematic Review of Pediatric and Adult Studies
Source: JMIR Mhealth Uhealth. 2021 Jul 8;9(7):e29619. doi: 10.2196/29619 (PMC8299344; doi:10.2196/29619)
Supplement: Multimedia Appendix 3 [file mhealth_v9i7e29619_app3.docx]

**Multimedia Appendix 3.** Additional results of the included studies in adult populations.

| **Source (Country)** | **Additional results** |
| --- | --- |
| Agarwal 2014 (India) | - 2,091 donors were recruited in an eight-month period - Average of 75.83% of phone calls were successful, out of which an average of 9.18% donated blood - 63% of in-house donations and 13% of total donations were recruited over the phone |
| Applebaum 2012 (USA) | - Higher total therapeutic alliance scores predicted decreased depressive symptoms - Higher task scores predicted decreased overall distress, depressive symptoms, avoidance symptoms, and reexperiencing symptoms - Higher bond scores predicted decreased depressive symptoms and reexperiencing symptoms |
| Bakitas 2015 (USA) | - No significant difference in quality of life (P=0.34), symptoms (P=0.09), mood (P=0.33) between early and late palliative care - Statistically greater Kaplan-Meier 1-year survival rate for early group than late group (P=0.038) - No significant difference in resource use for hospital visits (P=0.26), intensive care unit dates (P=0.49), emergency room visits (P=0.21), chemotherapy in the last 14 days (P=0.27), and home death (P=0.60) |
| Barcellona 2013 (Italy) | - Significantly higher number of blood checks in the home monitoring group compared to the conventional group - The percentage of missed INR checks was significantly higher during the conventional monitoring period - Significantly higher number of missed INR checks during the conventional monitoring period - There was a significant increase in time within the therapeutic range with home monitoring in the unstable group (P < .001), but there was no significant difference in the stable group - Self-testing is suitable and safe for oral anticoagulation management - No significant differences in number of hemorrhages or thrombotic events between the two groups |
| Blissit 2015 (USA) | - There were low rates of venous thromboembolism overall (1.1% and 1.8%) - Telephone group had a significantly lower number of clinic visits per year (P=0.00165) - No significant difference in rates of hospitalization, rate of significant bleeding, deep vein thrombosis, pulmonary embolism, cerebrovascular accident, and death from any cause between face-to-face and telephone groups - Statistically significant higher rate of any bleeding in face-to-face compared to telephone groups (P=0.0144) |
| Breen 2017 (Australia) | - Reported benefits included patient reassurance and empowerment, timely intervention when needed, and improved recall of side effects from chemotherapy - The system was more beneficial to patients with multiple side-effects - Suggested changes included language clarification and additional side-effect monitoring options - Patients fund the system easy to use alongside their daily routines |
| Burwick 2018 (USA) | - Median time to completion of an e-consult was two days - 96% of e-consults had a hemoglobin greater than 10 g/dL - 90% of e-consults had a creatinine less than 2 mg/dL - Median follow-up time after e-consult was 44 months - Mean progression rate of 1% per year - Six documented progression events |
| Cecchini 2016 (USA) | - Mean time to complete an e-consult by a hematologist was 14.5 minutes - Among patients who responded, 65% preferred an e-consult to a face-to-face visit - Among providers who responded, 100% were “satisfied” or “very satisfied” with e-consults - 18% reduction in face-to-face consults within the first 2 years of e-consult implementation (total number of consults increased during this time) |
| Clarke 2011 (Canada) | - 712 total telehealth encounters conducted - 83.6% encounters were conducted by medical oncologists - 15.7% encounters were conducted by genetic counselors - 0.7% encounters were conducted by medical geneticists - Most common telehealth appointment types were gastrointestinal cancer and lymphoma - Telehealth encounters were conducted by a total of 46 health care providers - A single medical oncologist conducted 58.7% of all telehealth appointments in 2009 - Local Health Areas with the highest number of oncology telehealth appointments were Kamloops (34.1%), Penticton (14.1%), Cranbrook (9.7%), Southern Okanagan (5.5%) |
| Compaci 2011 (France) | - 3592 phone calls, resulting in 989 interventions, were conducted - 950 cases of Grade 1 intervention - 39 cases of Grade 2 intervention - The nurse was able to provide follow-up and care management in 95% of cases - Compared to literature, implementation of Ambulatory Medical Assistance phone calls showed a lower incidence of secondary hospitalization, delayed treatment, reduced relative dose-intensity, toxic death, and red blood cell transfusion - More information is needed to assess the cost benefit of Ambulatory Medical Assistance telephone calls |
| Flannery 2009, (USA) | - 5,283 telephone calls conducted from 1,486 different patients over the span of 86 workdays - Seven telephone calls were received or made for every 10 scheduled appointments - Number of telephone calls was significantly higher on Mondays and in the mornings - 30% of telephone calls involved multiple reasons |
| Hung 2014 (Australia) | - Compared to usual care group, extended care group exhibited increases in protein intake (P=0.165), cognitive functioning (P=0.337), and social functioning (P=0.165) - Extended care group experienced less weight loss compared to baseline than usual care group (P=0.062) - Physical activity was not significantly different between groups - Usual care group reported a longer length of hospital stay compared to extended care group |
| Kirsh 2015 (USA) | - Rate of eConsults for hematology was 3.0 per 100 total consults - Within the first 3 months after an e-consult, there was a decreased likelihood of a subsequent face-to-face visit (P<0.001) - Within the first 3 months after an e-consult,, there was an increased likelihood of primary care visits (P<0.001) - Percentage of e-consults within one year of implementation rose for 28.5% to 44.4% - e-consult patients would have needed to travel an average of 72.1 potential miles per visit |
| Najafi 2017 (USA) | - 7 of 9 hospitalists (78%) and 7 of 11 consultants (64%) completed the survey - Hospitalists and consultants completing the survey agreed that e-consults were efficient and easy to use - All hospitalists completing the survey were satisfied with the quality of e-consults - 43% of consultants who completed the survey reported that they could provide high-quality consultation via e-consult - Top 5 specialties with the highest volume of e-consults were infection diseases, hematology, endocrinology, nephrology, and cardiology |
| Overend 2008 (Canada) | - 85% of patients completed the questionnaires - 82% of patients felt strongly that they could talk easily and openly with the nurse - 82% felt strongly that the nurse/doctor was able to understand their situation and provide them with satisfactory care - 71% disagreed, or were neutral, that it was necessary for a physician to examine them - 62% felt strongly that they would participate in a teleclinic again instead of travelling to see their oncologist - Mean distance that patients would normally have to travel to see an oncologist was 107 kilometers |
| Philip 2015 (USA) | - 16% increase in clinical pharmacy patient volume at the ambulatory care clinics after intervention (P= 0.011) - No significant difference between groups in time in therapeutic range, hospitalizations due to thromboembolic or bleeding events, work hours per prescription volume, project completion rates, or number of students precepted |
| Prochaska 2017 (Germany) | - Major and clinically relevant bleeding rates were higher in the regular care group than in the e-health service group - Thromboembolic event rate ratio of 1.5 comparing regular care to e-health service - Regular care showed more frequent rates of hospitalization and all-cause mortality - Increased frequency of home visits needed for e-health service compared to regular care (P=0.002) |
| Reid 2011 (Ireland) | - 7498 total calls were made to the chemotherapy telephone helpline in 2007 - 25.6% of calls occurred outside 8AM-4PM - Callers included patients (45.8%), lay carers (31%), and health care professionals (20.5%) - 35.2% of calls were regarding patients with multiple concerns or symptoms - 36.8% of calls resulted in patients being medically assessed |
| Skeith 2017 (Canada) | - 162 thrombosis eConsult cases completed - 47.5% of face-to-face consultations were avoided - 4.3% of e-consults resulted in a follow-up referral that was not already scheduled - Greater than 96% of primary care physicians highly rated the value of eConsult service |
| Syrjala 2011 (USA) | - 775 (58% of those eligible) participants consented and completed a baseline assessment - 57% participants required staff contact at least one time - Majority of the staff contact needed was for minor technical issues, delays in enrollment, or baseline assessment - Most participants initiated contact through email instead of the toll free line |
| Testa 2005 (Italy) | - No difference in time in therapeutic range between in anticoagulation clinics versus telemedicine use in general practitioner units - Telemedicine use in nursing homes showed a lower percent in therapeutic range compared to anticoagulation clinics. - No difference in major complications rates between telemedicine use in peripheral units and anticoagulation clinics - Patients showed a general improvement in their overall quality of life with the use of telemedicine |
| Woods 2000 (USA) | - 77 telemedicine sickle cell disease clinics held - Mean of 6.1 encounters per clinic - 466 total encounters among 128 sickle cell disease patients - With the use of telemedicine, the productivity of the clinic increased from 1413 to 1889 encounters per year - Rural outreach activity increased from 271 to 745 encounters per year - No significant difference in patient satisfaction scores between telemedicine and stand care |
| Woods 1999 (USA) | - No significant difference in patient satisfaction scores between telemedicine and standard care groups (p = 0.389) - Patients in the standard care group were more likely to offer positive open-ended comments than the telemedicine group (P=0.001) - There were 14/52 negative comments in the telemedicine group, generally due to confidentially, technology, and access concerns |
| Woods 1998 (USA) | - Mean clinic encounter time was 24 minutes - No significant difference in clinic encounter time between telemedicine and standard care groups - Mean compliance with scheduled clinic appointments was 76.6% |
| Wright 2007 (Canada) | - Patient’s adherence rate for follow-up visits was 87.5% - Patient’s immunosuppressive medications were successfully discontinued - Patient’s oral graft-versus-host disease symptoms were well-managed - Patient’s pain level was reduced to 0 - Patient indicated satisfaction with telehealth technology and reported that the ability to stay in his home significantly contributed to his well-being |
